# Retraction Note: *Turdoides affinis* mitogenome reveals the translational efficiency and importance of NADH dehydrogenase complex-I in the Leiothrichidae family

**DOI:** 10.1038/s41598-023-45261-6

**Published:** 2023-10-24

**Authors:** Indrani Sarkar, Prateek Dey, Sanjeev Kumar Sharma, Swapna Devi Ray, Venkata Hanumat Sastry Kochiganti, Renu Singh, Padmanabhan Pramod, Ram Pratap Singh

**Affiliations:** 1 National Avian Forensic Laboratory, Sálim Ali Centre for Ornithology and Natural History, Anaikatty, Coimbatore, Tamil Nadu 641108 India; 2 Central Avian Research Institute, Bareilly, Uttar Pradesh 243122 India; 3 Department of Life Science, Central University of South Bihar, Gaya, Bihar 824236 India

Retraction of: *Scientific Reports* 10.1038/s41598-020-72674-4, published online 01 October 2020

The Authors have retracted this Article.

After publication of the Article the Authors discovered errors in mitogenome extraction and data analysis, including manual curation of the mitogenome. As a result of these errors 14% of the reported mitogenome sequence, which is the main contribution of this Article, is inaccurate.

All Authors agree with the retraction and its wording.

